# Climate-driven spatial mismatches between British orchards and their pollinators: increased risks of pollination deficits

**DOI:** 10.1111/gcb.12577

**Published:** 2014-05-02

**Authors:** Chiara Polce, Michael P Garratt, Mette Termansen, Julian Ramirez-Villegas, Andrew J Challinor, Martin G Lappage, Nigel D Boatman, Andrew Crowe, Ayenew Melese Endalew, Simon G Potts, Kate E Somerwill, Jacobus C Biesmeijer

**Affiliations:** 1 School of Biology, Faculty of Biological Sciences, University of LeedsLeeds, LS2 9JT, UK; 2 School of Agriculture, Policy and Development, Reading UniversityReading, RG6 6AR, UK; 3 Department of Environmental Science, Aarhus UniversityRoskilde, 4000, Denmark; 4 School of Earth and Environment, University of LeedsLeeds, LS2 9JT, UK; 5 CGIAR Research Program on Climate Change, Agriculture and Food Security (CCAFS)Cali, DC, USA; 6 Food and Environment Research AgencySand Hutton, York, YO41 1LZ, UK; 7 Naturalis Biodiversity CenterPO Box 9517, RA Leiden, 2300, The Netherlands

**Keywords:** climate change, Maxent, perennial fruit, pollination services, range shifts, species distribution models

## Abstract

Understanding how climate change can affect crop-pollinator systems helps predict potential geographical mismatches between a crop and its pollinators, and therefore identify areas vulnerable to loss of pollination services. We examined the distribution of orchard species (apples, pears, plums and other top fruits) and their pollinators in Great Britain, for present and future climatic conditions projected for 2050 under the SRES A1B Emissions Scenario. We used a relative index of pollinator availability as a proxy for pollination service. At present, there is a large spatial overlap between orchards and their pollinators, but predictions for 2050 revealed that the most suitable areas for orchards corresponded to low pollinator availability. However, we found that pollinator availability may persist in areas currently used for fruit production, which are predicted to provide suboptimal environmental suitability for orchard species in the future. Our results may be used to identify mitigation options to safeguard orchard production against the risk of pollination failure in Great Britain over the next 50 years; for instance, choosing fruit tree varieties that are adapted to future climatic conditions, or boosting wild pollinators through improving landscape resources. Our approach can be readily applied to other regions and crop systems, and expanded to include different climatic scenarios.

## Introduction

Numerous examples show how predicted climate change will affect land suitability and crop yields; and how increased frequency and intensity of extreme weather events can increase fluctuations in crop yields (Schmidhuber & Tubiello, 2007; Lobell *et al*., 2008; Challinor *et al*., 2010; Hawkins *et al*., 2013). Minimizing the negative effects that these fluctuations have on food security, requires an understanding of the effects of climate change on the resilience of crop growth and yield (Fraser *et al*., 2013) and, where relevant, of crop-pollinator systems. Eighty-seven of the leading global food crops, accounting for about 35% of the global production, benefit from animal pollination, in particular from insects (Klein *et al*., 2007); yet, pollination is rarely accounted for in projections of the impacts of climate change on crop yields.

The importance of insect pollinators has been valued at €153 billion to agricultural production (Gallai *et al*., 2009) worldwide. The documented declines in insect pollinators (Biesmeijer *et al*., 2006; Potts *et al*., 2010; Carvalheiro *et al*., 2013) may therefore threaten food security, which in turn may lead to an increased demand for agricultural land (Aizen *et al*., 2009). Climate change, together with land-use intensification and the spread of alien species and diseases, is one of the main anthropogenic pressures on insect pollinators (Vanbergen & The Insect Pollinators Initiative, 2013). Several authors have investigated how climate change can affect plant-pollinator interactions (e.g. Devoto *et al*., 2007; Memmott *et al*., 2007). Recent work by Kuhlmann *et al*. (2012), for instance, has highlighted how climate-induced range shifts of dominant bee species are likely to affect specialized plant-pollinators mutualisms, with negative consequences for the reproduction of these plants. Other studies have looked at specific crop-pollinator systems, examining the cost of replacing pollination service (Allsopp *et al*., 2008), the predicted decline in suitable bee habitat due to climate change (Giannini *et al*., 2012), and the potential environmental suitability of nearby regions allowing the persistence of crop-pollinators mutualisms (Giannini *et al*., 2010, 2011).

Understanding how climate change will affect crop-pollinator systems helps highlight areas potentially vulnerable to pollinator shortage, or predict potential geographical mismatches between crops and their pollinators. Similarly, such information can be used to identify areas suitable for the persistence of both the crop and its wild pollinators, or direct local interventions to boost pollination service, thereby strengthening food security.

In this study, we examined the impact of projected climate change on the distribution and likelihood of occurrence of commercial orchards and their pollinators in Great Britain (GB). Commercial orchards (orchards hereafter) occupy about 19 000 ha in UK, which equates to nearly 13% of the total area dedicated to the production of fruit and vegetables. Of the area used for fruit production, orchards occupy 65.5%, with the remainder used for the production of soft fruits, such as strawberries. The major products of orchards in the UK are apples, pears, plums and cherries, with dessert apples alone accounting for nearly 28% of the planted area and having a net value of £70 million annually (Department for Environment, Food & Rural Affairs, 2013). Bees and hoverflies are the predominant pollen vectors for these plants (Klein *et al*., 2007). To ensure marketable fruits, therefore, it is important that the activity of pollinators overlaps spatially and temporally with the flowering period of the fruit trees.

Here, we used the projected climate for 2050 from the SRES A1B Emissions Scenario (Nakićenović *et al*., 2000) to estimate the nation-wide environmental suitability for orchard species and their pollinators. Recent trajectories of greenhouse gas emissions are higher than those considered under the SRES A1B (Raupach *et al*., 2007), so the projections used here may be interpreted as conservative estimates of climate change. We used present day distributional data to characterize the climatic conditions most favourable to the orchard species and to their pollinators. We excluded land-cover/land-use information, since its distribution cannot readily be predicted with sufficient certainty beyond the current time. We then projected the potential distribution of orchards and pollinators, on the basis of the climate projected for 2050, and derived a relative measure of pollination service available to orchards for present and future conditions. This approach allowed us to highlight changes in environmental suitability which may threaten the persistence of the orchard-pollinator system, and identify geographical mismatches between orchards and pollinators potentially affecting pollination service provision. We used this information to suggest appropriate intervention measures which could be used to mitigate against future loss of pollination services to orchards.

## Materials and methods

### Overview

We used the species distribution model (SDM) Maxent, (Phillips *et al*., 2006; Aguirre-Gutiérrez *et al*., 2013; Polce *et al*., 2013) to predict the distributions of orchards and pollinators in relation to climatic conditions. Pollinator and orchard data were collected using different methodologies, thus requiring different modelling approaches; we describe these datasets in the following subsections ‘Pollinator data’ and ‘Orchard data’ respectively. Details of the climate data used to characterize the environmental space of orchards and pollinators are provided in the subsection ‘Climate data’. We modelled pollinator species individually, using available records to predict current potential distribution and then future projections. We modelled future distribution of orchard species as an entire category since information on orchard composition was not available from the current agricultural survey data. The model settings for pollinator species and orchards are described separately, under ‘Distribution models’. We then describe how we used the outputs from the SDMs to identify:

1. The climatic predictors that contributed most to the final models (subsection ‘Contribution of predictors’).
2. How the climatic predictors for the pollinators and orchards are projected to change in 2050 (subsection ‘Similarities between current and predicted climate’).
3. An index of relative pollinator availability to orchards, which we used as a proxy for potential pollination service (subsection ‘Pollinator availability’).

## Pollinator data

We used presence-only sightings of wild bees and hoverflies recorded in GB within the period 2000–2010 (‘Bees, Wasps and Ants Recording Society’, BWARS, http://www.bwars.com/; ‘Hoverfly Recording Scheme’, HRS, http://www.hoverfly.org.uk/). On the basis of literature (Free, 1993; Marini *et al*., 2012) and knowledge gained by our team members during the past years of pollinator-related field projects (e.g. http://www.reading.ac.uk/caer/Project_IPI_Crops/project_ipi_crops_index.html; http://www.step-project.net/; http://www.alarmproject.net/alarm/; all accessed February 2014), we selected 22 species of wild bees and 8 species of hoverflies, known to be visitors of fruit trees and therefore potential pollinators of orchard crop flowers in GB. The spatial resolution of the records varied from 100 m^2^ to 4 km^2^. We aggregated all sightings for each species on the 25 km^2^ grid (5 by 5 km cells) and removed any duplicate records, so that for each species, there was at the most one entry per grid cell. The number of available records per bee species ranged from 26 to 2096 (mean ± SD = 650 ± 580; median = 471); records per hoverfly species ranged from 150 to 1981 (mean ± SD = 1032 ± 616; median = 1033). Pollinator species and numbers of records are listed in the Data S1 (Table S1 in Data S1).

## Orchard data

The current distribution of orchards was derived from the 2010 Defra June Agricultural Survey (http://www.defra.gov.uk/statistics/foodfarm/landuselivestock/junesurvey/junesurveyresults/). Orchards included areas of at least 1 ha, planted with top fruit such as apples, cherries, pears, plums and nuts (walnuts and hazelnuts mainly); their distribution was originally mapped on a grid of 2 × 2 km cells (‘tetrads’) and included information on the extent of the orchards within each tetrad. We superimposed a 5 × 5 km grid onto the crop tetrads, proportionally allocating each tetrad's orchard extent to the overlapping 5 km grid cell(s). The final extent of the orchards within each cell was the sum of the proportional extent from all tetrads intersecting the cell. Of the 9726 grid cells used to represent GB, around 14% contained orchards (1354), with a total mapped extent greater than 12 200 ha. The difference between the Defra figures for orchards and the actual mapped hectares are due to insufficient spatial information for some of the orchard fields to be mapped.

## Climate data

We used total annual precipitation and monthly minimum and maximum temperature to derive a set of environmental descriptors commonly used in species distribution models (e.g. Hijmans & Graham, 2006; Warren *et al*., 2010; Warren *et al*., 2013; Wolmarans *et al*., 2010): growing degree days greater than 5 °C (GDD5, used only for crop), calculated following Sork *et al*. (2010); and 19 bioclimatic variables (Hijmans *et al*., 2005, 2011). This choice reflected the need to satisfy two main criteria: the same predictors needed to be available for both the present and the future projections; they needed to be relevant for the modelled group. The three input variables were obtained from UKCP09 (http://www.metoffice.gov.uk/climatechange/science/monitoring/ukcp09/). Baseline data for pollinator distribution models were made of the 25 km^2^ gridded monthly averages for the decade 1990–1999, while for the orchard distribution model, we used gridded data for the 30 year period 1977–2006 (the most recent available complete 30 year period). We used a longer time series for orchard crops, to reflect the longer life cycle of fruit trees compared to insect pollinators. Future projections of monthly averages were derived from the UKCP09 projections (Murphy *et al*., 2010) for the SRES A1B storyline (‘Medium’ Emissions Scenario, as referred in the UKCP09 report). We used the 30 year period from 2040 to 2069; we will refer to the baseline data as the ‘Present’ and to the future projections as the ‘M2050’. These data are located on a rotated-pole grid with a spatial resolution of approximately 25 by 25 km. We rescaled them to the 5 × 5 km British National Grid, to match the orientation and resolution of the baseline data. Additional information on this dataset and details of the rescaling procedure are provided in the Data S1 (Data S1, Material and Methods, ‘Climate data for future projections’).

All the variables were computed within R (R Development Core Team, 2011). To minimize colinearity between predictors (Guisan & Thuiller, 2005), subsets were created for pollinators and orchards. For pollinators, due to lack of a general set of commonly used variables, we used Jolliffe's Principal Component Analysis with the rejection method ‘B2’ and *λ*_0_ = 0.70 (Jolliffe, 1972, 1973); we reduced the original set to six predictors (Table 1). For orchards, we based the choice on literature (Thuiller, 2004; Termansen *et al*., 2006; Sork *et al*., 2010; Franklin *et al*., 2013; Warren *et al*., 2013), and we selected five predictors (Table 1). The Pearson's correlation between the selected crop and pollinator variables is reported in the Data S1 (Data S1, Material and Methods, ‘Correlation between selected climatic predictors’, Tables S2 to S5).

**Table 1:** Variables used for orchards and pollinators distribution modelling. The table shows the final set of predictors used to model the distribution of orchards (ODM) and pollinators (PDM). The selection of predictors was based on several criteria, including their use in published literature and minimizing multicollinearity

Original code	Definition	Abbreviation	Model
Bio03	^a^Isothermality	Isoth	PDM
Bio04	Temperature Seasonality (SD × 100)	TSeasSD	ODM
Bio06	Min Temperature of Coldest Month	mTCM	ODM
Bio07	Temperature annual range	TAR	PDM
Bio08	Mean Temperature of Wettest Quarter	MTWQ	ODM
Bio09	Mean temperature of driest quarter	MTDQ	PDM; ODM
Bio11	Mean temperature of coldest quarter	MTCQ	PDM
Bio15	Precipitation seasonality (Coefficient of variation)	RainSeasCV	PDM
Bio18	Precipitation of Warmest Quarter	RainWQ	ODM
Bio19	Precipitation of coldest quarter	RainCQ	PDM

Isothermality = Mean diurnal range/Temperature annual range × 100. Isothermality quantifies how large is the day-to-night temperature oscillation in comparison to the summer-to-winter oscillation, with 100 representing a site where the diurnal temperature range is equal to the annual temperature range.

## Distribution models

### Pollinator distribution models

Detailed settings for the Maxent pollinators’ distribution models (PDM) follow Polce *et al*. (2013) and are summarized in the Data S1 (Data S1, Material and Methods, ‘Pollinator distribution model’). Model training and testing was performed through 10-fold cross-validation, and ‘10th percentile of training presence’ was used as a threshold to convert probability of occurrence into binary predictions (‘presence-absence maps’). We chose this threshold since it retains as suitable environmental conditions, those characterizing 90% of the training locations, thus excluding records that were found at the extreme of the species’ suitable environment. We assumed unlimited dispersal capability for each species, but we restricted the predicted presence to areas where all 10 model runs had predicted ‘presence’; we assigned average probability of occurrence (*p*) to these areas, and ‘absence’ outside them.

We assessed the models using the Area Under the Receiver Operating Characteristic Curve (AUC), which, despite known assumptions and limitations (Termansen *et al*., 2006; Austin, 2007), is commonly used as a threshold-independent measure of model performance within SDMs. With presence-only data such as the pollinators’ sightings, the maximum achievable AUC is <1 (Wiley *et al*., 2003) so standard thresholds for evaluating goodness of fit do not apply. Instead, we followed Raes & ter Steege (2007) and we compared the average AUC value of each species PDM (AUC_PDM_) with the average AUC value of a set of null models (AUC_NM_) where species records were replaced by randomly chosen locations. We expected AUC_PDM_ > AUC_NM_.

### Orchard distribution models

After running Maxent models using different feature classes (i.e. including different possible relationships between species data and climate variables from linear to hinge to quadratic) to predict present orchards distributions, we retained the models that used hinge features, which were then used to predict orchards’ future probability of occurrence. For the orchard distribution model (ODM), Maxent was trained on 75% sample points, and the remainder was used for testing. This procedure was repeated 10 times. We used areas where at least seven model runs had predicted presence (based on the ‘10th percentile of training presence’), to indicate suitable conditions for crop growing under the M2050 scenario, and assigned to these areas the average probability of occurrence obtained from the 10 model runs. We used a more relaxed criterion for orchards than pollinators (7 vs. 10 model runs to indicate presence), to account for the fact that orchards are a managed resource and so can overcome some of the barriers which would prevent colonization and establishment of wild organisms such as the pollinators that were modelled.

## Contribution of different predictors to distribution models

The contribution of each predictor to the final PDMs and ODM was derived from the drop in AUC observed after permuting the values of each variable with those of the background, with larger drops indicating that the model depended heavily on that variable (Data S1, Material and Methods, ‘Contribution of different predictors’). Average and confidence interval for the observed drops were derived through 10 000 bootstrap replicates.

We used a linear mixed effects model (Pinheiro *et al*., 2013) to test if the contribution of different predictors differed between pollinator species and/or model runs. We used predictors as fixed factors and model run as a random factor. Model run was nested within species and group (bees or hoverflies) when analysing the results from the PDM. Multiple pairwise comparisons of different predictors were then performed using Tukey's *post hoc* test for a general linear hypothesis (Hothorn *et al*., 2013).

## Similarities between current and predicted climate

The PDM and ODM were required to predict conditions not sampled in training data. Computing the similarity between conditions at training locations and conditions where predictions are to be made can be done within Maxent, through Multivariate Environmental Similarity Surfaces (MESS) (Elith *et al*., 2010). MESS measure the similarity of any given point to a set of reference points, for each model predictor. The lowest similarity obtained for that point is used as the point's MESS. Negative values indicate conditions that are outside the range of references values, while positive values indicate greater similarity to the set of reference points, with 100 assigned to a point not novel at all (i.e. having a predicted value within the range of reference points). In addition to mapping the MESS across the region of interest (GB), an accompanying map also showed, at any given location, the variable that drove the MESS. We used these two maps to spatially quantify predicted climatic changes.

## Pollinator availability

We used pollinator availability (PA) as a proxy for pollination service. Pollinator availability resulted from the contribution of each species probability of occurrence predicted by the Maxent model. We assumed that all pollinator species are equally efficient in pollinating orchard flowers. For each grid cell, where the presence of orchards was predicted or observed, PA was:

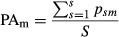
1 Where: PA_m_ = pollinator availability to the orchards in grid cell *m,* resulting from all pollinator species; *p*_sm_ = Maxent probability of occurrence for species *s* on cell *m*;*S *= total number of pollinator species. Eqn  is loosely based on Lonsdorf *et al*. (2009) and Polce *et al*. (2013), with the main difference being that the weighted term allowing pollinators to reach an orchard located on neighbouring cells is excluded, as the model resolution is coarser than the typical pollinator foraging distance.

We used Eqn to derive the PA where:

1. Orchards are currently present: the baseline PA;
2. Orchards are predicted to occur based on the M2050 future scenario: to assess whether the most suitable areas for fruit trees are also suitable for their pollinators;
3. Orchards are currently present, but climatic conditions are those predicted for M2050: to derive the difference in PA between the present and the M2050 scenario, assuming that fruit trees can continue to persist where currently present, despite changes in climatic conditions.

## Results

### Climate and bioclimatic variables

Plotting the climatic variables used for pollinators and crop models highlighted any distributional difference in the predictor values between Present and M2050 (Figure S1 in Data S1). We identified three patterns: (i) No major differences in range of values or distribution; (ii) Similar distribution with systematic shift; (iii) Change in the mean and the shape of the distribution. Table 2 groups the predictors according to the observed change.

**Table 2:** Qualitative assessment of the changes observed between present and future climatic predictors. The table groups the climatic predictors according to the changes observed between present and M2050 projections. Three main types of changes were identified (in *Italics*). Climatic predictors are defined in Table 1

Observed change	Characteristics of the future projections
*No major differences*	
MTCQ	
TAR	
RainWQ	
*Similar distribution with systematic shift*	
mTCM	Warmer
MTWQ	Warmer
TSeas	Twice as big
*Change in the mean and the shape of the distribution*	
Isoth	Expanded to include lower values, indicating areas with a greater difference between the diurnal temperature range and the summer-to-winter oscillations
MTDQ	Changed from a normal distribution towards a bimodal distribution, with a small peak towards lower temperature, and a greater one towards temperature 5 degrees higher than then present mean
RainSeas	Changed from having two peaks and a trough to having one peak, around the values currently characterized by the trough (i.e. 25%), indicating a greater number of sites with similar rainfall pattern

**Figure 1:**
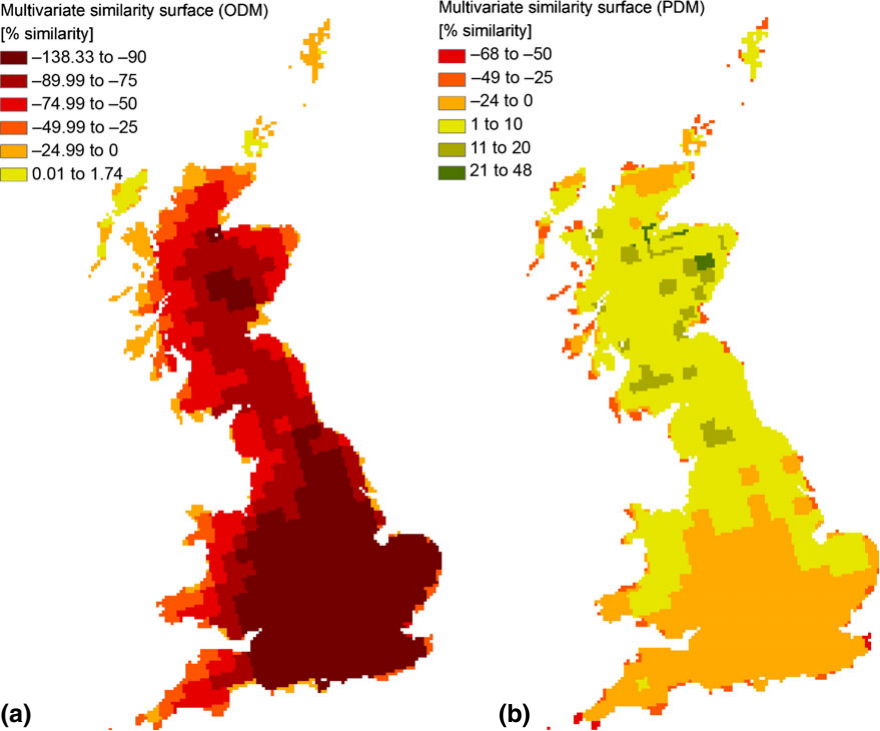
Multivariate environmental similarity surface (MESS) from (a) orchards and (b) pollinators’ distribution models (ODM and PDM respectively) The two maps summarize the environmental similarity between the present parameter space used during model training (reference value), and the parameter space used to project model results (the future M2050 scenario), for (a) orchards and (b) pollinators. Negative values indicate conditions that are outside the range of references values, while positive values indicate greater similarity to the set of reference points; 100 would indicate a point not novel at all.

**Figure 2:**
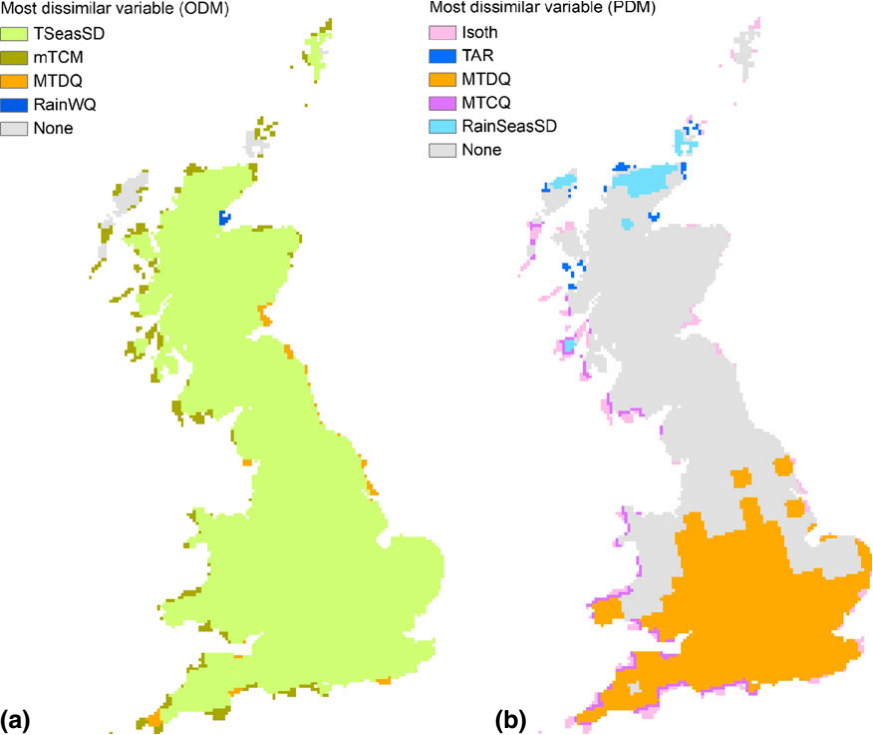
Most dissimilar variable (MoD) from (a) orchards and (b) pollinators distribution models (ODM and PDM respectively). The two maps show the MoD based on the multivariate environmental similarity surface derived from the predictors used to model (a) orchards and (b) pollinators distribution and mapped in Fig. 1. Grey areas indicate that the distribution of values predicted for the M2050 future is within the range of values observed in present time. It is important to note that MoD is defined over the entire range of values for each predictor, and therefore cannot be used to assess whether the predicted climatic conditions at a particular location differ from the baseline. Variables are defined in Table 1.

The environmental similarities between the parameter space used during model training and the parameter space used for the M2050 projections are summarized in Fig. 1 and Fig. 2. The two maps in Fig. 1 show the MESS: they have to be read in relation to the predictors used to build the model, hence the differences between the orchards- and pollinators-MESS. The pattern of the orchards-MESS reveals a latitudinal gradient, with dissimilarity increasing southwards, and coastal areas generally holding environmental conditions within the present range (Fig. 1a). With between species subtle differences in the decimal values, the pollinators-MESS showed a zoned similarity, with novel climate concentrated in the South and often along the coast (Fig. 1b).

The most dissimilar variable at any given locality across the orchards- and pollinators-MESS is shown in Fig. 2. The most dissimilar variable from the orchards-MESS was Temperature Seasonality (TSeasSD) in almost the entire region (92%), while the area that had not experienced shifts in the parameter space summed to only 2% (Fig. 2a). The most dissimilar variable derived from the pollinators-MESS showed a different pattern: the vast majority (55%) of the area was characterized by climatic conditions within the present range. For areas characterized by novel climatic conditions (novel in relation to the training points), Mean Temperature of the Driest Quarter (MTDQ) was the most dissimilar variable, covering the Southern part of the region, for 36% of the total extent (Fig. 2b).

## Pollinator and crop distribution models

### Model performance

Pollinators distribution models provided a significantly better fit than expected by chance alone for all the species: AUC of model testing ranged from 0.57 to 0.92 (average AUC = 0.72), while null model AUC ranged from 0.48 to 0.51 (average AUC = 0.49) (Figure S2 in Data S1). A similar pattern was observed for the model training AUC. ODMs rendered average model testing AUC = 0.80, varying from 0.79 to 0.82, which is considered within the range of useful applications (Swets, 1988).

### Contribution of different predictors

There was a significant difference in the permutation importance of the different predictors resulting from the PDMs. Temperature Annual Range (TAR) was the most important predictor (bootstrap mean importance: 30.7%); Mean Temperature of the Coldest Quarter (MTCQ) followed next (26.8%) and was significantly less important than TAR (Tukey's *post hoc* test: *P *=* *0.01). Isothermality (Isoth) and Mean Temperature of the Driest Quarter (MTDQ) were, equally (*P* = 0.36), the least important predictors (Figure S3, Table S6 and Table S7 in Data S1).

Predictors’ importance was consistent across different runs of the ODMs: Temperature Seasonality (TSeasSD) was the most important predictor (bootstrap mean 39.0%), followed by Minimum Temperature of the Coldest Month (mTCM, bootstrap mean 35.6%) (Figure S4 in Data S1). Multiple pairwise comparisons between the predictors confirmed a ranking of decreasing importance (Figure S4, Table S8 and Table S9 in Data S1). Given the potential effects of mTCM on the fulfilment of the chill hours, we inspected Maxent models built with this variable alone: probability of orchard occurrence was greatest for 1 ≤  mTCM ≤ 2 °C and decreased nearly symmetrically outside this interval (Figure S5 in Data S1).

## Future projections

### Orchard model

**Figure 3:**
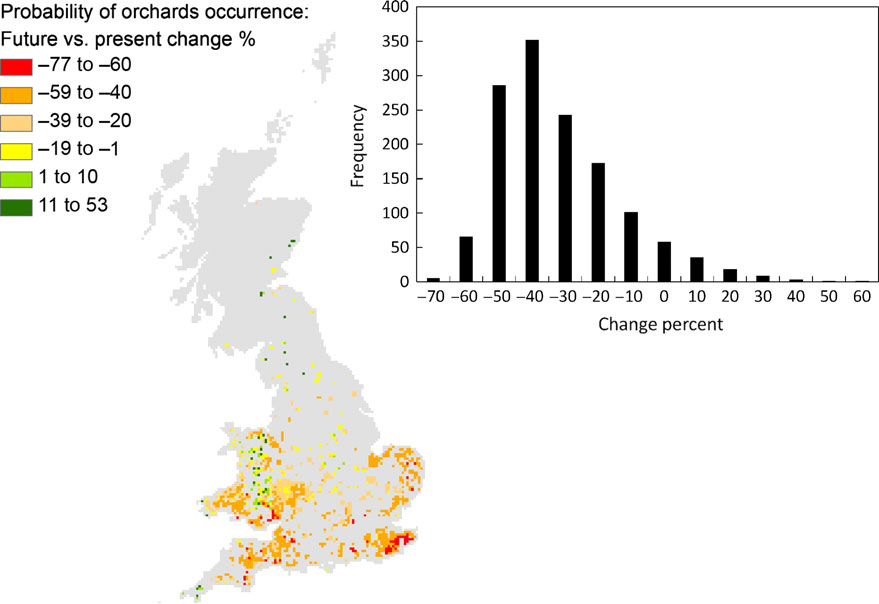
Per cent change in probability of occurrence at the localities currently occupied by orchards. The map shows the spatial distribution of the change in probability of occurrence (*p*) between the future scenario and the present conditions, for the localities currently occupied by orchards. The difference in *p* between the two periods (*p*_Future_*–p*_Present_) was converted to percentage. The histogram shows the frequency of the change, and it highlights that negative changes are expected to be more frequent than positive changes, implying that most of the areas currently occupied by orchards are expected to become less suitable.

**Figure 4:**
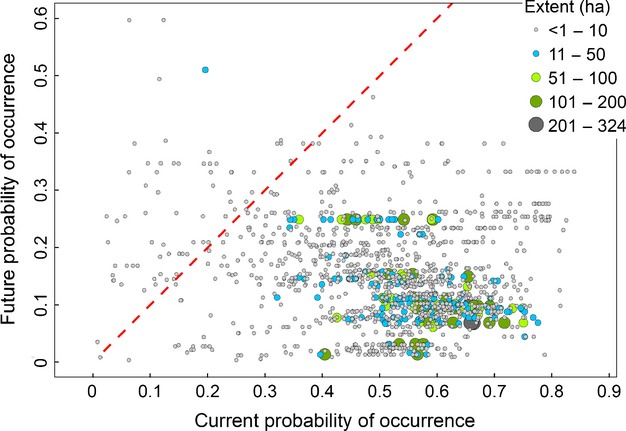
Current orchards’ extent in relation to current and predicted probability of occurrence based on climate suitability. Current extent measured in hectares is mapped with proportional symbols, using five intervals from <1 ha to 324 ha. The map suggests that most of the largest orchards are currently in areas where the model has predicted probability of occurrence (*p*) > 0.4, while the same orchards will be in areas with future *p *<* *0.3 and in most cases *p *<* *0.2. The red line marks where current *p *= future *p* and highlights that the predicted environmental suitability for larger orchards is lower than the current one.

Per cent change between M2050 and present in probability of occurrence (*p*) for existing orchards ranged from −77.5% to +53.4% (mean ± SD: −36.9% ± 18.7%, Fig. 3). Negative changes were more frequent than positives (Histogram in Fig. 3). At the locations of existing orchards, present probability of occurrence ranged from 0.01 to 0.84 (mean ± SD: 0.52 ± 0.15), with the largest crop parcels found in areas with *p *>* *0.40. In the M2050 scenario, however, the predicted probability of occurrence for the current locations of the largest orchards decreased to *p *<* *0.30 and in most cases *p *<* *0.20, indicating a change in the climatic conditions at the sites currently used for orchards (Fig. 4). The pattern shown in Fig. 4 was confirmed by testing the correlation between orchard size and probability of occurrence. We found that size of orchards and probability of occurrence were positively correlated under present climatic conditions (*ρ *= 0.153), but negatively correlated for the M2050 scenario (*ρ *= −0.233). Both correlations were significant based on 9999 samples with replacement taken at random from the two distributions (Figure S6 in Data S1).

**Figure 5:**
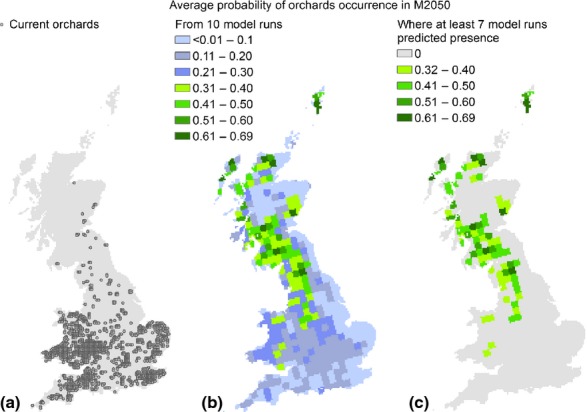
Current orchards locations and future probability of occurrence. From left to right: the first map (a) shows the locations of the current orchards. The second map (b) shows the orchards’ average probability of occurrence (*p*) in the M2050 future, as predicted from 10 model runs. The third map (c) indicates the average *p* for areas where at least seven of the 10 model runs have predicted crop presence, based on the threshold defined in the main text.

The predicted probability of occurrence for orchards in M2050 from the average of 10 model runs ranged from 0.01 to 0.69, with the highest values in the Western part of GB, in areas currently not occupied by orchards. If we only include regions where at least seven of the 10 model runs have predicted crop presence, the minimum probability of occurrence becomes 0.32, while the maximum remains unchanged (Fig. 5). These regions, chosen by the vast majority of the model runs, were taken as the most likely locations for fruit tree production under the M2050 scenario (i.e. those with most suitable climate; soil and other growing conditions may still be limiting) for subsequent estimates of the service provision. In addition, the minimum probability of occurrence found for these regions (0.32) was within 2 SD of the mean obtained at the localities of orchards based on current predictions (mean–2SD: 0.22).

### Pollinator models

**Figure 6:**
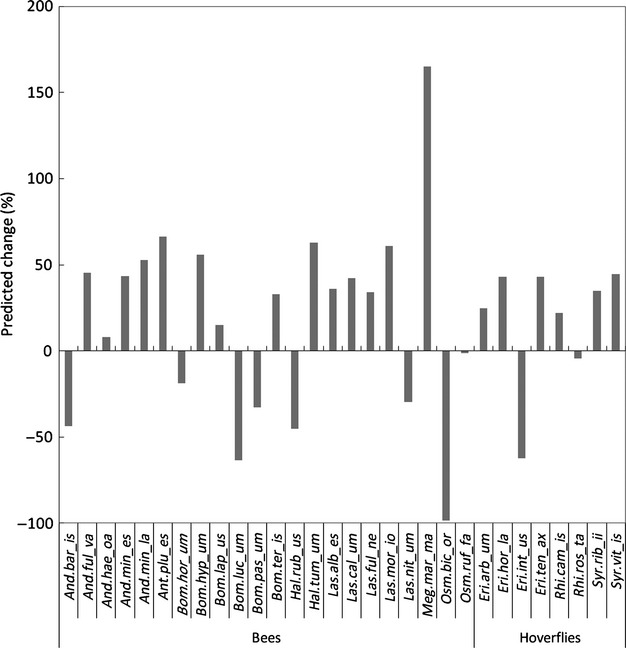
Predicted change in species’ range. Per cent change is obtained by comparing each species’ predicted future range to its current range, under a universal expansion hypothesis (i.e. no barriers to expansion). Negative values indicate loss of suitable areas (i.e. range contraction) while positive value indicate gain of areas (i.e. range expansion). Within each group (bees, hoverflies), species are listed alphabetically, with the dot separating the first three letters of the genus from the first three and last two letters of the species.

Pollinators distribution models predicted a range expansion for 20 species and range contraction for 10 species. Range expansion varied from 8% to 165% (*Andrena haemorroa* and *Megachile maritima* respectively); range contraction varied from 1% to 99% (*Osmia bicornis* and *O. bicolor* respectively) (Fig. 6). The overall mean and median were 18% and 33% respectively, indicating a greater change in the direction of range expansion than in range contraction.

**Figure 7:**
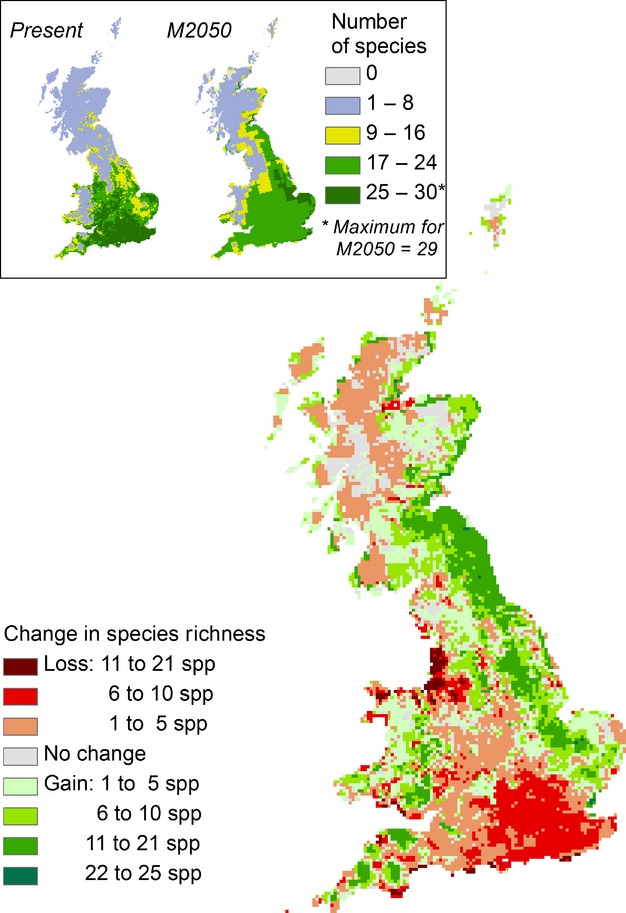
Change in species richness between current and future predictions. The maps in the inset show the predicted number of pollinator species, based on the Maxent models. Species richness (SR) was predicted within the range of 0–30 for present, and 0–29 for the M2050 scenario. SR is displayed using intervals and coloured using blue shades for areas with lower SR (i.e. diversity cold spots) to green shades for areas with higher SR (i.e. diversity hot spots). The larger map shows the change in SR, resulting from comparing the predicted SR for the two periods. Gains of species are mapped in green, with darker shades indicating greater species gain. Losses of species are mapped in red, with darker shades indicating greater species loss. Grey indicates areas where SR was not predicted to change.

The areas with greatest species richness (SR) were predicted to occur in a large part of Southern GB for present time (SR = 25–30); for the M2050 only a small area in the Eastern part of the country was predicted to reach a similar richness (SR = 25–29) (Fig. 7). In addition, comparing the predicted SR for the two periods, revealed an area of greatest species loss in the Western part of GB (11–21 species lost), and an extensive area with the opposite trend along the East coast (11–21 species gained). The largest areas where SR did not change were in the Northern part of GB, mainly in the Eastern and Western part of Scotland.

### Pollinator availability

**Figure 8:**
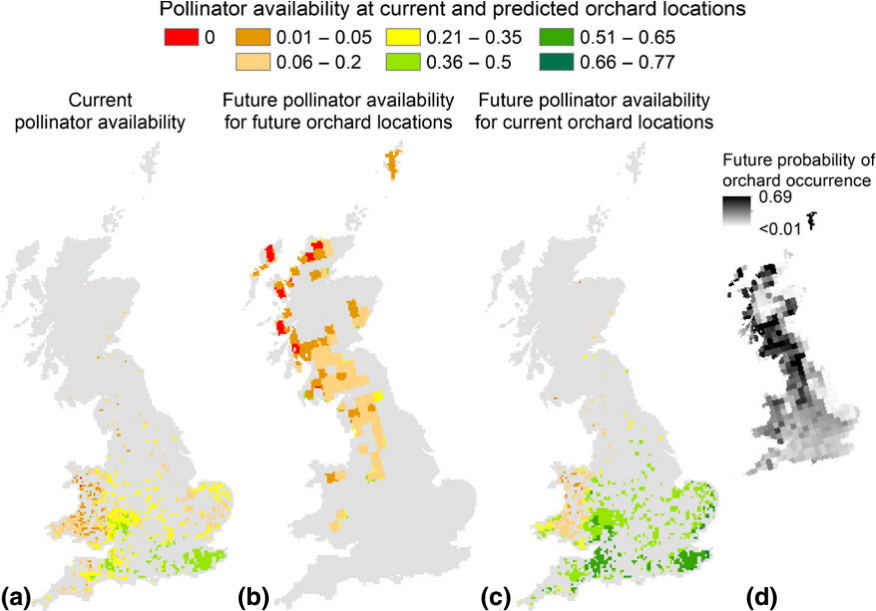
Pollinator availability for different scenarios of orchards distribution and climatic conditions. Pollinator availability (PA) is used as a proxy for pollination service, and measured with a relative index from 0 to 1. PA is mapped from red to green using intervals. Red is used only to indicate areas where PA is predicted to be 0 (i.e. where pollinators are predicted to be absent). The first map (a) shows the PA currently available to orchards. The second map (b) shows the future PA for areas most suitable to orchards based on the M2050 scenario. The third map (c) shows the PA predicted where orchards are currently planted, but based on the pollinator availability for M2050. The smaller map (d) shows the probability of occurrence (*ρ*) for orchards, based on the M2050 climatic conditions. Darker areas indicate greater *p*: this map suggests that, under future climatic conditions, *ρ* for areas where orchards are currently planted is predicted to be low.

**Figure 9:**
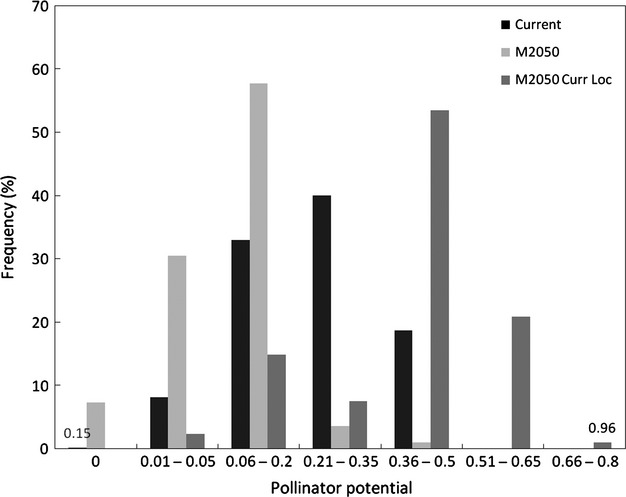
Distribution of classes of pollinator availability. Bars show the distribution of classes of pollinator availability (PA) mapped in Fig. 8, according to their per cent frequency. Frequency was defined as the number of grid cell characterized by a certain PA. Black bars represent current PA; light grey show the PA for areas where orchards have the greatest probability of occurrence based on the future scenario M2050; dark grey show the PA based on climatic conditions predicted for M2050 but for areas where orchards are currently planted.

The present pollination availability (PA) ranged from 0 (absence of pollinator) to 0.48 (Fig. 8a and Figure S7 in Data S1): complete absence of pollinators for crops was predicted for <1% of the horticultural areas (Fig. 9) and was localized mainly in the Western part of Wales; 73% of the area was predicted within the range 0.06 ≤ PA ≤ 0.35, while the highest class of PA (0.35 < PA ≤ 0.5) was predicted for 19% of the areas, mainly in the Southern part of GB. The PA to orchards for the locations predicted by the M2050 scenario ranged again from 0 to 0.48 (Fig. 8b and Figure S8 in Data S1), but its distribution differed from present. Absence of PA characterized 7% of the horticultural areas (Fig. 9) and was mainly localized along the North-west coast (Scotland); the majority of the area (89%) was predicted to have 0.01 ≤ PA ≤ 0.2, along the central and Western part of the country. The highest class of PA was only predicted for 1% of the potential future horticultural areas, which was made up of a few isolated fragments. The last scenario shows the PA available for M2050, but at the locations where orchards are currently planted. PA ranged from 0.01 to 0.77 (Fig. 8c and Figure S9 in Data S1) indicating a change in the pollinator distribution from the present patterns. All the orchards were predicted to be exposed to pollinators, and thus to potentially benefit from their service. The class of PA corresponding to the highest currently predicted (0.36–0.5) occupied 53% of the area (Fig. 9), while it was 1% for present conditions. An even greater PA, namely 0.51 < PA < 0.8 was predicted for 22% of the area, generally corresponding to the regions currently receiving the greatest PA (the Southern part of GB). However, looking at the future orchards, *p* shown in Fig. 8d reveals that these areas are characterized by low *p*, indicating that the climatic conditions may not favour crop growth.

## Discussion

It has been widely documented that shifts in species ranges are correlated with climatic change (Parmesan & Yohe, 2003; Root *et al*., 2003; Chen *et al*., 2011). By acting on the distribution and survival of single species, climate change is likely to affect ecosystem functions and, as a result, the provisioning of ecosystem services. In this study, we examined the potential consequences of climate change for pollination services as provided by an array of 22 bee and 8 hoverfly species known to be frequent visitors of orchards in Great Britain. Changes in climatic conditions can affect plant-pollinator interaction networks in several ways (Hegland *et al*., 2009), for instance by causing phenological mismatches (Burkle *et al*., 2013) or spatial mismatches (Schweiger *et al*., 2008). Here, we focused on potential geographical mismatches, and showed that under future climate scenario, suitable conditions for orchards and orchard pollinators may not overlap, threatening pollination service. In particular, we examined the potential distribution of pollinators and orchard species grown in GB, based on the SRES A1B Emissions Scenario climatic projections. We used a relative measure of pollinator availability as an indication of potential pollination service, since quantifying service delivery in absence of pollinator abundance data cannot be done. Using the environmental suitability for wild pollinators, as a relative measure of potential pollination service is a commonly adopted approach (Lonsdorf *et al*., 2009; Lautenbach *et al*., 2011; Polce *et al*., 2013; Zulian *et al*., 2013), in absence of sufficient information to parameterize the relation between pollinator availability and yield.

Pollinator species differ in their efficacy to pollinate flowers (e.g. Bischoff *et al*., 2013; Castro *et al*., 2013; Garratt *et al*., 2014). In addition, altered phonologies due to climate change may result in temporal mismatches between the availability of the most effective pollinators and the onset of flowering, with potential negative consequences on plant reproduction success (Rafferty & Ives, 2012) and service provision. Although approaches to estimate and compare pollinators’ performances have been discussed (Ne'eman *et al*., 2010; King *et al*., 2013), there remain practical and theoretical difficulties to apply the proposed methods over large geographical regions and to many pollinator species. Thus, in deriving pollinator availability, we did not take into account species’ identity and we assumed all species be equally efficient in pollinating orchard flowers.

We chose orchards as they represent a major GB fruit crop, and they include top fruits of global economic importance, such as apples. The distribution of orchards is limited to locations having suitable soils, climate and socio-economic conditions. For examples, apples and other fruits trees are known to be vulnerable to frost occurring during bloom stage: the projected climate warming, therefore, raises concerns that the bloom stage might advance in time and coincide with periods where frost spells can happen, thus threatening the quality and possibly the production of fruits (Eccel *et al*., 2009). Of the predictors used to model orchard distribution in our study, Temperature Seasonality presented the greatest mismatch between present and future, with projections shifted towards greater variability. We cannot assert at this stage that this will directly threaten fruit production, but greater variability may increase the risk of sharp temperature variations, such as the occurrence of frost spells in periods otherwise characterized by milder temperatures, e.g. during flowering. In addition, fruit trees benefit from bud dormancy, which is triggered by a period of exposure to cold weather: the predicted rise in Minimum Temperature of Coldest Month (mTCM) could interfere with the fulfilment of the chill hours per year, potentially affecting the production of leaves, flowers and subsequently fruit. Inspecting Maxent models built exclusively with this variable provide some support to this hypothesis. While current mTCM is most commonly between 1 and 2 °C, this will increase to 4 °C, with peaks up to 8 °C, in M2050.

Of the predictors used to model pollinator distribution, Mean Temperature of the Driest Quarter was the one with the greatest shift, both in terms of mean and shape; but for the majority of the country none of the predictors moved outside the present climatic range. In interpreting this pattern, we must stress that change only refers to the range of values of the predictors, and not to their geographical location; in other words, a location will be mapped as ‘No change’ if the projected values for all predictors have changed, but they have all remained within the range of values observed for present time. The results from the PDMs projected that locations with greatest pollinator richness would shift north-east, suggesting a similar shift in environmental conditions most suitable to pollinators. For Europe, Ohlemüller *et al*. (2006) have already shown a prevailing north-east shift in the climatic conditions analogous to the 1931–1960 period. For much of the global land shifting climate has been projected to be greater than 1 km yr^−1^ over the 21st century (Diffenbaugh & Field, 2013), potentially posing alarming challenges for terrestrial ecosystems. The results of the PDMs assume unlimited dispersal of pollinators and predicted range expansion to occur more frequently than range contraction. Indeed, some species are likely to track such changes. The bumblebee, *Bombus hypnorum*, arrived in SE England less than 15 years ago and since then has reached Scotland. However, if areas of similar climate are farther than the species’ dispersal distance, colonization and persistence may not be possible (Thuiller, 2004; Ohlemüller *et al*., 2006), and more species would shrink their range. This risk would be further enhanced by other pressures acting on the pollinators, such as habitat fragmentation and degradation, parasites and alien species (Vanbergen & The Insect Pollinators Initiative, 2013), none of which was considered here. Looking at the species that are already predicted to experience range contraction, some of them, like *Osmia* and *Bombus* spp., are known to be efficient pollinators of orchard trees, and of apples in particular (Delaplane & Mayer, 2000). Therefore, geographical mismatches between these species and orchards might have noticeable effects on pollination service provision. There could be expansion of orchard pollinators from the continent into GB, although this element was not included in our study; there could also be additional pollination supply from managed pollinators (e.g. honeybees), although the capacity to utilize honeybees for additional pollination services is primarily independent of climate.

Solely based on climatic projections, the most suitable environmental conditions for orchards shifted north-west, although probability of occurrence for these areas never reached the maxima obtained for the present. Since our projections were only based on climate, however, they must be read with caution: much of the areas identified as suitable for orchards in M2050 occur in uplands that may not be suitable for fruit tree cultivation owing to soil type and topography. In addition, the pollinator availability predicted for these areas was for the vast majority ≤0.2, in contrast with present predictions which showed larger areas with 0.2 ≤ PA ≤ 0.5. These results suggest that, over the next 50 years, the most suitable areas for orchards may not be characterized by pollinator availability as high as now. Furthermore, while the present distribution of orchards largely overlapped areas with the highest pollinator richness, future predictions showed a geographical mismatch between areas most suitable to orchards and areas richest in pollinator species. Pollinator diversity has been observed to increase fruit set in several crop systems (Klein *et al*., 2003; Hoehn *et al*., 2008; Garibaldi *et al*., 2013) and buffer negative effects of extreme weather events such as strong winds (Brittain *et al*., 2013). Adequate pollination could still be possible by a few species of wild bees with high numbers of individuals, but such a community would be more vulnerable to stressors and stochastic variation. Landscape management to increase pollinator diversity and abundance in these areas of future orchard production could be implemented to improve the stability of pollination services, such as preservation of seminatural landscapes or increasing pollinator habitat and forage resources (Ricketts *et al*., 2008; Scheper *et al*., 2013), or additional inputs from managed pollinators might become necessary to achieve optimal yields.

In contrast, the areas currently occupied by orchards are predicted to become even more suitable to pollinators in M2050. Under this scenario, however, due to unfavourable conditions the predicted probability of the occurrence of orchards will decrease. New top fruit varieties could be developed with future climatic conditions in mind, particularly breeding for resistance to those factors identified in this study as key to driving the shift in orchard distribution, namely Temperature Seasonality and Minimum Temperature of the Coldest Month.

In this study, we have used species distribution models and climate projections to derive the environmental suitability for the orchard-pollinator system in Great Britain, under different scenarios. Due to the characteristics of the pollinator data, we used a relative measure of pollinator availability which cannot (yet) be translated into units of service delivery (Maes *et al*., 2012). Our approach, however, detected a geographical mismatch in climatic suitability for orchards and pollinators, which may potentially lead to low pollination service provision, unless production is moved towards more (climatically) suitable north-westerly areas. However, we found that wild pollinator availability may be preserved and possibly enhanced in areas already used for orchards. The implications of trading off between wild pollinator availability and lower climatic suitability need further research. In particular, methods of boosting wild pollinators through improving landscape resources (Scheper *et al*., 2013), supplementing wild pollination service with managed pollinators, or choosing fruit tree varieties that are adapted to changed climatic conditions may provide a combination of adaptation options to support top fruit production in GB over the next 50 years. The methods underlying our study can be applied to other regions and crop systems, and expanded to include different climatic scenarios. Some of the most urgent challenges that need to be addressed, are the inclusion of other factors limiting future crop cultivation (e.g. soil type), and the translation of the relative measure of pollinator availability into units of service delivery.

## Supporting Information

Data S1.Climate-driven spatial mismatches between British orchards and their pollinators: increased risks of pollination deficits.
