# Supplementary material for: Climate-driven spatial mismatches between British orchards and their pollinators: increased risks of pollination deficits
Source: Glob Chang Biol. 2014 May 2;20(9):2815–28. doi: 10.1111/gcb.12577 (PMC4285753; doi:10.1111/gcb.12577)
Supplement: Supplementary file 1 — Data S1.Climate-driven spatial mismatches between British orchards and their pollinators: increased risks of pollination deficits. [file gcb0020-2815-sd1.pdf]

Climate-driven spatial mismatches between British orchards and their pollinators: increased risks of pollination deficits.

Supporting Information

Material and Methods

Pollinator data

Table S 1 lists the wild insect pollinator species used to predict the potential pollinator availability to orchard crop flowers in Great Britain. Available records indicate the number of sites of known presence, mapped on 5 by 5 km grid cells. The honeybee *(Apis mellifera*) was excluded due to the difficulties of separating records from managed colonies from those of wild colonies.

Climate data for future projections

Monthly averages for future projections were derived from the daily data generated by the HadRM3-PPE-UK experiment (Murphy *et al.*, 2010), run at the UK Met Office Hadley Centre (MOHC). The dataset contained the outputs from an ensemble of eleven variants of the MOHC Regional Climate Model (HadRM3). The HadRM3 model run from 1950-2099 and was used to dynamically downscale global climate model results, as part of the climate change experiments carried out for the UK Climate Projections report (UKCP09). The data were obtained online after registration (http://badc.nerc.ac.uk/home/). For our study, we used the unperturbed model variant of the HadRM3-PPE-UK experiment (identified by the name HadRM3Q0).

The HadRM3-PPE-UK data are located on a grid characterised by a rotated-pole and a spatial resolution of approximately 25 by 25 km. We used the accompanying metadata to convert the rotated grid into a regular grid of latitude and longitude. To match the resolution of the baseline data, we then rescaled the data to a 5 by 5 km British National Grid, giving to each of the 5 by 5 km grid cell the value of the coarser 25 by 25 km grid.

Table S 1: Wild insect pollinator species visiting orchards and number of available records

Wild pollinator species	Available records
**Bees**	
Andrena barbilabris	169
Andrena fulva	331
Andrena haemorroa	652
Andrena minutuloides	68
Andrena minutula	431
Anthophora plumipes	295
Bombus hortorum	1388
Bombus hypnorum	698
Bombus lapidarius	1446
Bombus lucorum	1706
Bombus pascuorum	2096
Bombus terrestris	1318
Halictus rubicundus	378
Halictus tumulorum	534
Lasioglossum albipes	328
Lasioglossum calceatum	724
Lasioglossum fulvicorne	243
Lasioglossum morio	510
Lasioglossum nitidisculum	26
Megachile maritima	70
Osmia bicolor	129
Osmia bicornis (*O. rufa*)	766
**Hoverfly**	
Eristalis arbustorum	1200
Eristalis horticola	461
Eristalis intricarius	629
Eristalis tenax	1590
Rhingia campestris	1377
Rhingia rostrata	150
Syrphus ribesii	1981
Syrphus vitripennis	866

Correlation between selected climatic predictors

Table S 2 shows the Pearson's correlation between the variables used to predict orchards distribution. Variables were selected from the original set of 20 climatic predictors, using literature (Franklin *et al.*, 2013, Sork *et al.*, 2010, Termansen *et al.*, 2006, Thuiller, 2004, Warren *et al.*, 2013). The values refer to present conditions, which for orchards corresponded to the 30-year period from 1977 to 2006. Variables are defined in Table 1 of the main text.

Table S 2: Pearson's correlation between present climatic predictors used to model orchards distribution

	TSeasSD	mTCM	MTWQ	MTDQ	RainWQ
**TSeasSD**					
**mTCM**	-0.03				
**MTWQ**	0.41	0.64			
**MTDQ**	0.14	0.64	0.30		
**RainWQ**	-0.58	-0.33	-0.52	-0.23	

Table S 3 shows the Pearson's correlation between the variables used to predict orchards distribution, based on the UKCP09 projections for the Medium emission scenario. The Medium emissions scenario corresponds to the A1B storyline of the IPCC's Special Report on Emissions Scenarios (Nakićenović *et al.*, 2000). We used the 30-year period from 2040 to 2069 ("M2050"). These variables were selected on present-day conditions using literature (see Table S 2) and are defined in Table 1 of the main text.

Table S 3: Pearson's correlation between future climatic predictors used to model orchards distribution

	TSeasSD	mTCM	MTWQ	MTDQ	RainWQ
**TSeasSD**					
**mTCM**	0.87				
**MTWQ**	0.04	0.36			
**MTDQ**	-0.80	-0.73	0.26		
**RainWQ**	-0.66	-0.49	0.37	0.81	

Table S 4 shows the Pearson's correlation between the variables used to predict pollinators' distribution. We used Jolliffe's Principal Component Analysis with the rejection method "B2" and λ_0 = 0.70 (Jolliffe, 1972, Jolliffe, 1973), to select a set of variables that minimised multicollinearity, from the original 19 bio-climatic predictors. The values refer to present conditions, which for pollinators corresponded to the 10-year period from 1990 to 1999. Variables are defined in Table 1 of the main text.

Table S 4: Pearson's correlation between present climatic predictors used to model pollinators' distribution

	Isoth	TAR	MTDQ	MTCQ	RainSeasCV	RainCQ
**Isoth**						
**TAR**	0.34					
**MTDQ**	0.16	-0.26				
**MTCQ**	0.01	0.04	0.17			
**RainSeasCV**	0.00	-0.43	0.42	0.00		
**RainCQ**	0.06	-0.62	0.32	-0.30	0.68	

Table S 5 shows the Pearson's correlation between the variables used to predict pollinators' distribution, based on the predictions for the Medium Emission Scenario (SRES A1B storyline), for the M2050 period. These variables were selected on present-day conditions using PCA (see Table S 4) and are defined in Table 1 of the main text.

Table S 5: Pearson's correlation between future climatic predictors used to model pollinators' distribution

	Isoth	TAR	MTDQ	MTCQ	RainSeasCV	RainCQ
**Isoth**						
**TAR**	0.87					
**MTDQ**	0.04	0.36				
**MTCQ**	-0.80	-0.73	0.26			
**RainSeasCV**	-0.66	-0.49	0.37	0.81		
**RainCQ**	0.03	-0.23	-0.27	-0.02	0.11	

Pollinator distribution models

Detailed settings for the Maxent pollinators' distribution models (PDM) follows Polce *et al.*(2013) . Models were set to run with *Hinge* features and with modified values of prevalence, to reflect the differences in number of available sightings at any resolution, from the original databases. The background was chosen to reflect the non-random distribution of the species' sightings in relation to the environmental range available for Great Britain. It corresponded to all localities where crop pollinators had been recorded (Phillips *et al.*, 2009).

Contribution of different predictors

The contribution of each predictor to the final Maxent model was measured using the permutation importance. Within Maxent, permutation importance is determined for each variable by randomly permuting the values of the variable among the presence and background training points and evaluating the resulting decrease in training AUC. The drop in AUC is then normalised to percentage allowing comparison across models; a larger percentage (a larger drop) indicates that the original model depended heavily on that variable. Average and confidence interval for the importance of the different predictors were derived through 10,000 bootstrap replicates.

Results

Climatic and bioclimatic variables

Each chart in Fig. S 1 shows the distribution of predictors' values for present (in black) and future M2050 (in grey) climatic conditions. The predictors are the ones used to model pollinators and orchards distribution. They are defined in Table 1 of the main text. The *y*-axis provides an estimate of the number of grid cells characterised by each predictor's value.

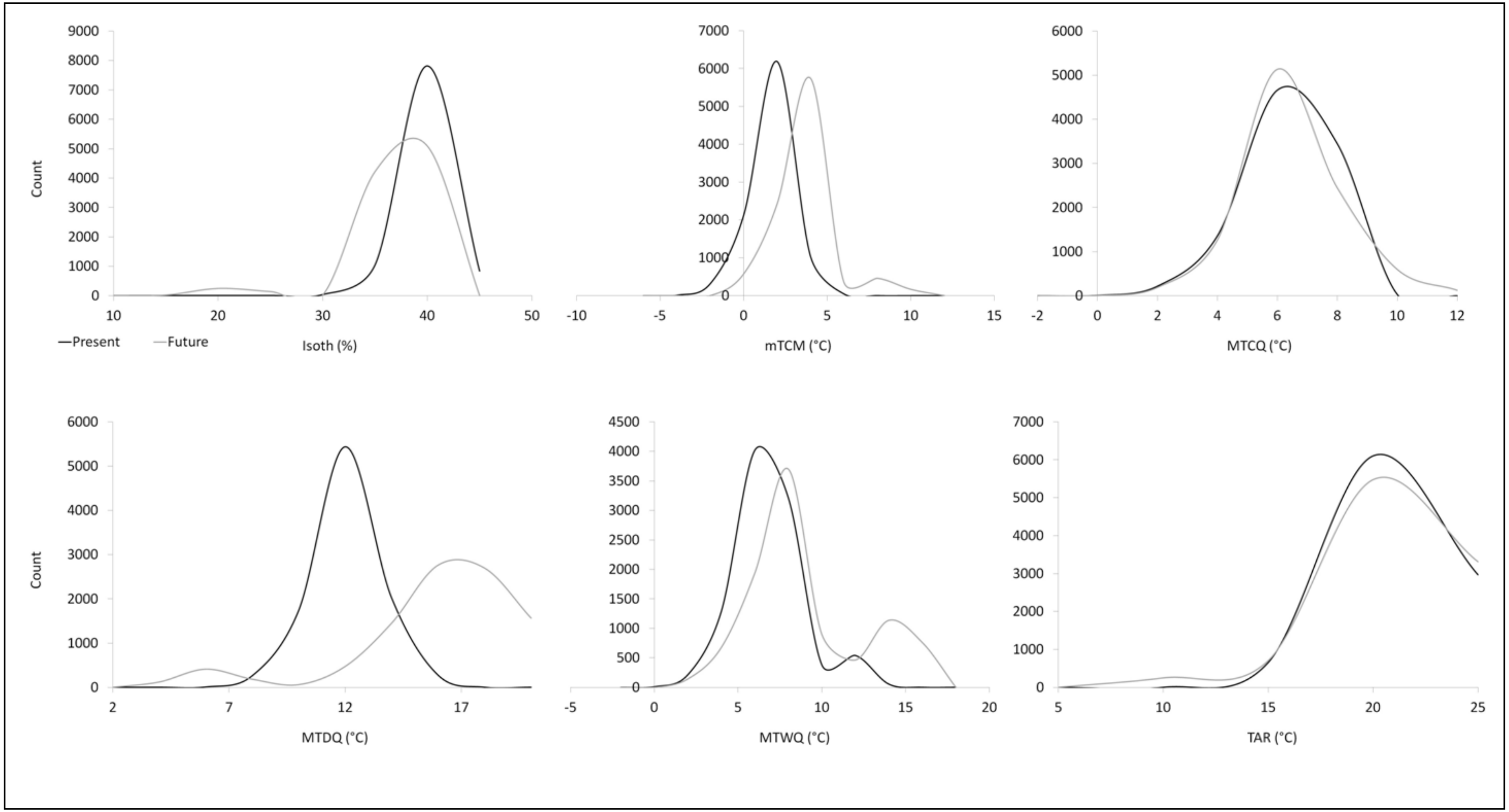

Figure S 1: Distribution of the predictors' values across GB, for present and future (continuing on next page)

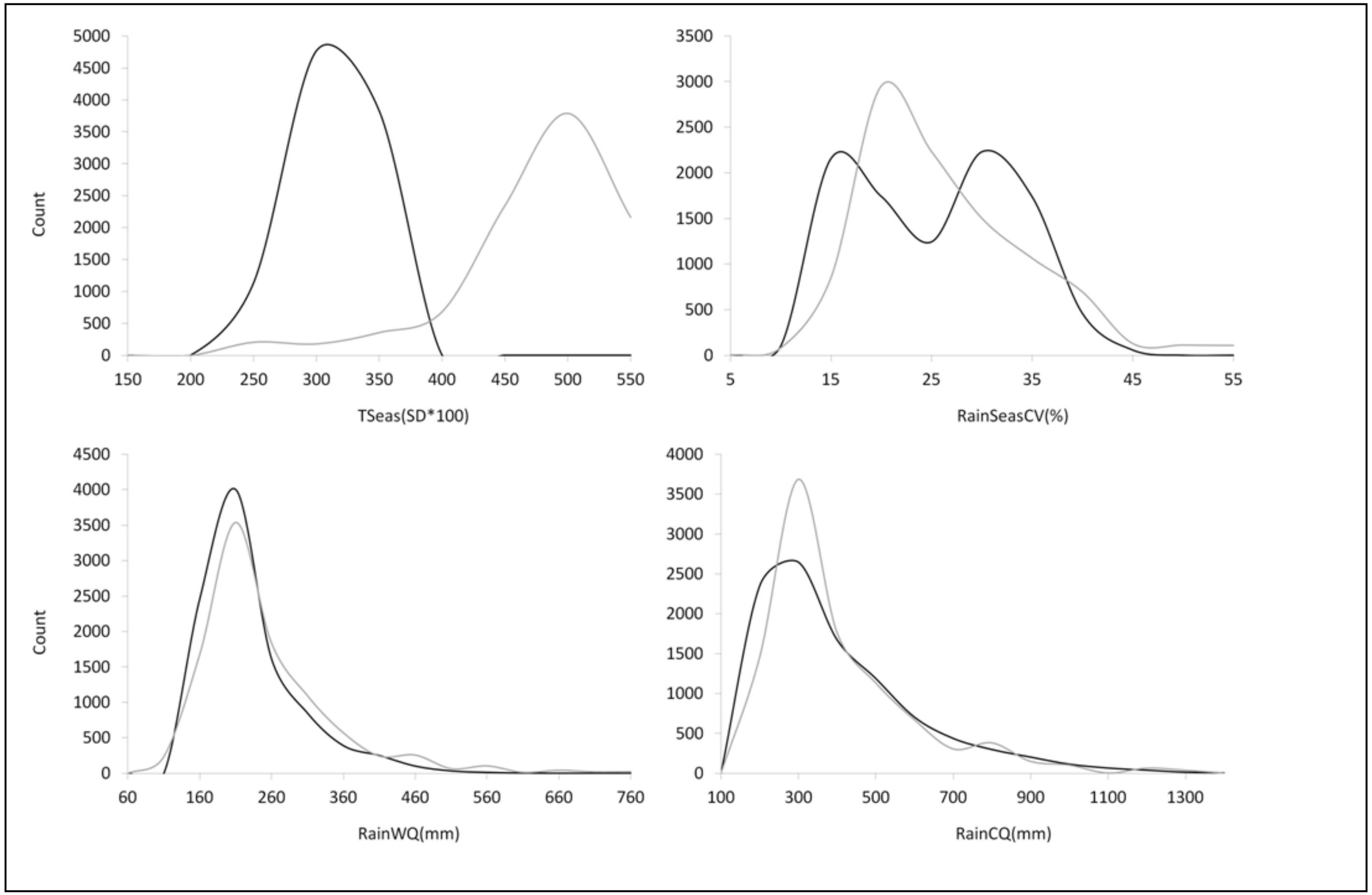

Figure S 1 (continuing from previous page): Distribution of the predictors' values across GB, for present and future

Model performance

Figure S 2 show the model performance for the pollinator distribution models, as measured by the the AUC of model testing. Error bars show the standard deviation of the null models (10 sets for each pollinator species, each modelled with 10-fold cross-validation). The number of available records is used to plot different species along the *x*-axis.

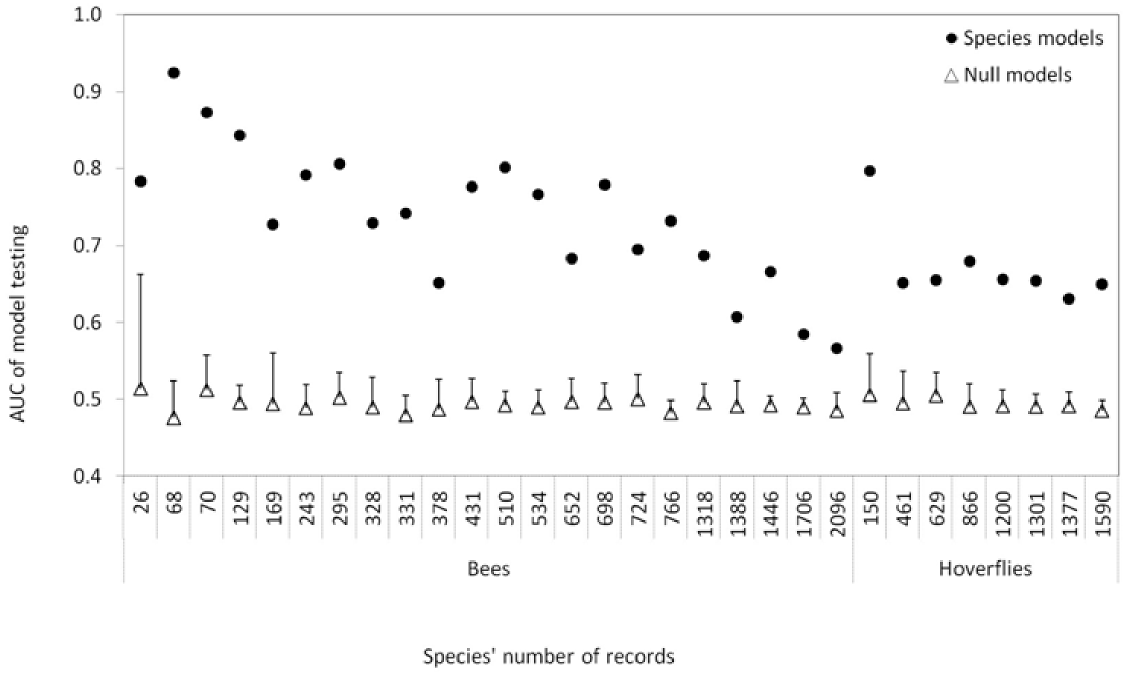

Figure S 2: Performance of the calibrated PDMs against performance of the null models

Contribution of different predictors

Figure S 3 shows the contribution of the different predictors used for PDMs, measured by the arithmetic and bootstrap mean of each predictor's importance, pooled across pollinator species. Confidence interval shows the 95% biased-corrected accelerated percentile, based on 10000 replicates. *TAR* was significantly more important than the other predictors, whilst *Isoth* and *MTDQ* were equally the least important. The significance of multiple pairwise comparisons was tested using Tukey's post-hoc test (Table S 6 and Table S 7). Predictors are defined in Table 1 of the main text.

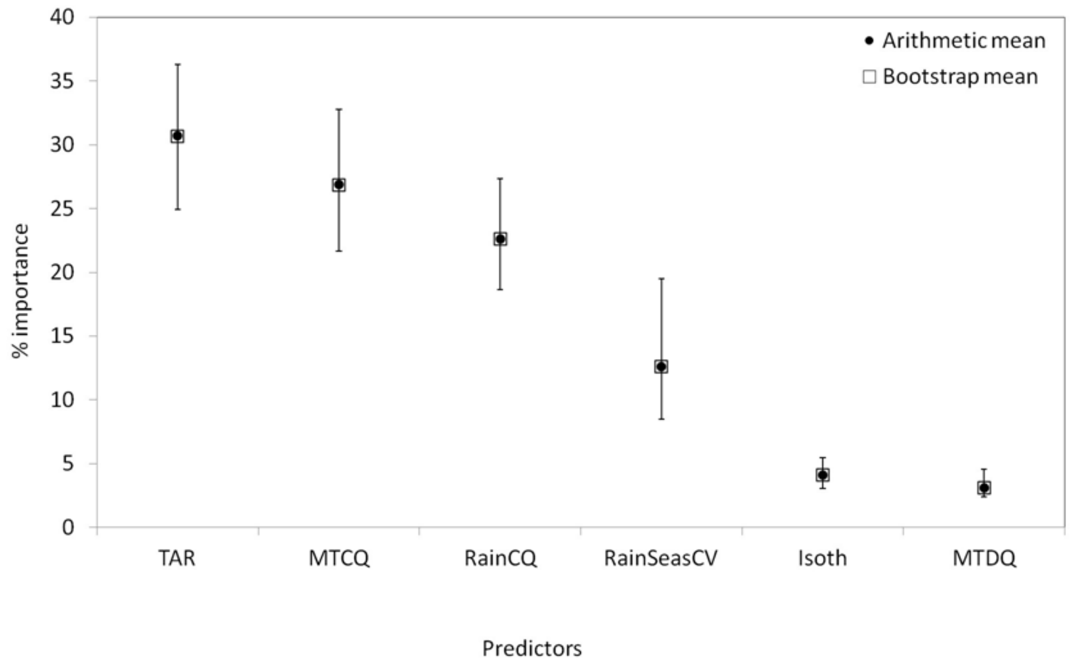

Figure S 3: Importance of predictors used for PDMs

Table S 6 shows the significance of the differences in permutation importance of the six predictors used for the pollinator distribution models. We used linear mixed effects model, with *model run* nested within *species* and *group* (bees or hoverflies) as a random factor, and *predictor* as fixed effect. Before the analysis, we applied an angular transformation to the permutation importance percent, to improve normality of residuals. We used *Isoth* as a baseline. Predictors are defined in Table 1 of the main text. Fixed effects only are shown here.

Table S 6: Difference in the importance of predictors used for PDMs

	Estimate	SE	DF	t	P
**Intercept**	0.186	0.009	1495	19.948	< 0.001
**MTCQ**	0.336	0.013	1495	25.400	< 0.001
**MTDQ**	-0.026	0.013	1495	-1.961	0.050
**RainCQ**	0.295	0.013	1495	22.320	< 0.001
**RainSeasCV**	0.132	0.013	1495	9.957	< 0.001
**TAR**	0.380	0.013	1495	28.757	< 0.001

Table S 7 shows the significance of the pairwise comparisons of means, for the different predictors used in PDMs. Multiple comparisons were assessed with Tukey's test, using general linear hypothesis test (Hothorn *et al.*, 2013). These results and those in Table S 6 show that *TAR* was the most important predictor, significantly more important than all others (e.g. *MTCQ*, which followed next). On the opposite end, with equal importance, *MTDQ* and *Isoth* were the least important predictors. Predictors are defined in Table 1 of the main text.

Table S 7: Multiple comparisons of means, for the importance of predictors used in PDMs

Linear hypothesis	Estimate	SE	z value	Pr(>|z|)
MTCQ - Isoth == 0	0.336	0.013	25.400	<0.001
**MTDQ - Isoth == 0**	**-0.026**	**0.013**	**-1.961**	**0.365**
RainCQ - Isoth == 0	0.295	0.013	22.320	<0.001
RainSeasCV - Isoth == 0	0.132	0.013	9.957	<0.001
TAR - Isoth == 0	0.380	0.013	28.757	<0.001
MTDQ - MTCQ == 0	-0.362	0.013	-27.362	<0.001
RainCQ - MTCQ == 0	-0.041	0.013	-3.080	0.025
RainSeasCV - MTCQ == 0	-0.204	0.013	-15.443	<0.001
**TAR - MTCQ == 0**	**0.044**	**0.013**	**3.357**	**0.010**
RainCQ - MTDQ == 0	0.321	0.013	24.281	<0.001
RainSeasCV - MTDQ == 0	0.157	0.013	11.918	<0.001
TAR - MTDQ == 0	0.406	0.013	30.718	<0.001
RainSeasCV - RainCQ == 0	-0.163	0.013	-12.363	<0.001
TAR - RainCQ == 0	0.085	0.013	6.437	<0.001
TAR - RainSeasCV == 0	0.248	0.013	18.800	<0.001

Figure S 4 shows the contribution of the different predictors used for ODMs, measured by the arithmetic and bootstrap mean of each predictor's importance, from different runs of the orchard distribution model. Confidence interval s show the 95% biased-corrected accelerated percentile, based on 10,000 replicates. *TSeasSD* was significantly more important than all other predictors. The significance of multiple comparisons was tested using Tukey's post-hoc test (Table S 8 and Table S 9). Predictors are defined in Table 1 of the main text.

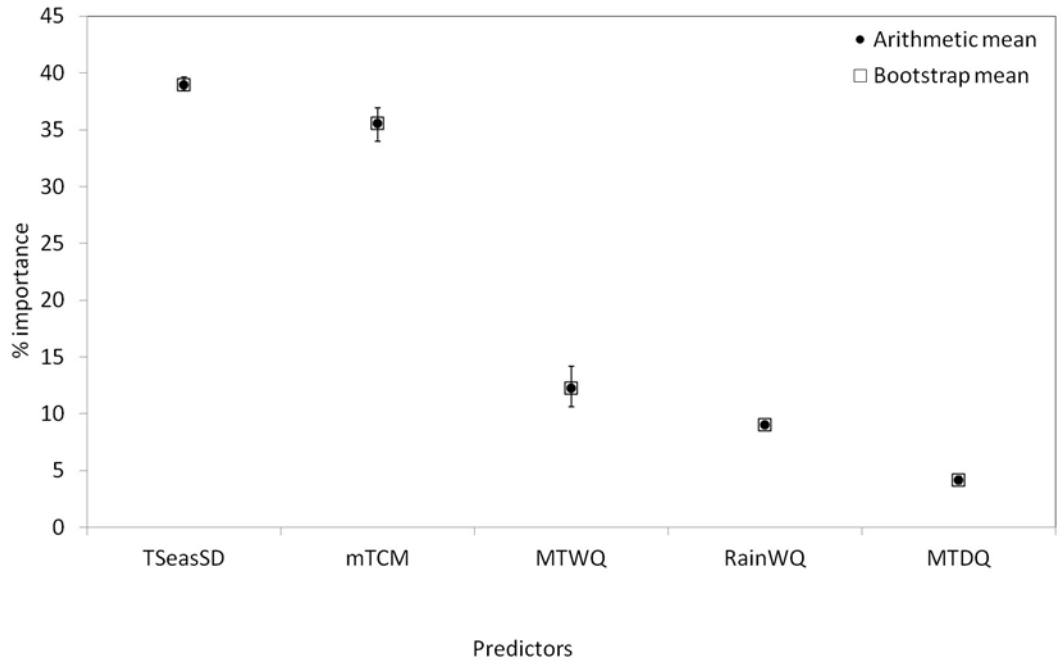

Figure S 4: Importance of predictors used for ODM

Table S 8 shows the significance of the differences in permutation importance of the five predictors used for the crop distribution models. We used linear mixed effects model with *model run* as a random factor and *predictor* as fixed effect. Before the analysis, we applied an angular transformation to the permutation importance percent, to improve normality of residuals. We used *Temperature Seasonality* as a baseline. Predictors are defined in Table 1 of the main text. Fixed effects only are shown here.

Table S 8: Difference in the importance of predictors used in ODM

	Estimate	SE	DF	t	P
**Intercept**	0.674	0.008	36	84.793	0.000
**mTCM**	-0.035	0.011	36	-3.108	0.004
**MTWQ**	-0.319	0.011	36	-28.343	0.000
**MTDQ**	-0.469	0.011	36	-41.759	0.000
**RainWQ**	-0.368	0.011	36	-32.770	0.000

Table S 9 shows the significance of the pairwise comparisons of means, for the different predictors used in ODM. Multiple comparisons were assessed with Tukey's test, using general linear hypothesis test (Hothorn *et al.*, 2013). These results and those obtained from the mixed effects model in Table S 8 show that *TSeasSD* was significantly more important than all other predictors, including *mTCM* which followed next. The remaining predictors followed in a significantly decreasing importance, according to the rank shown in Figure 5 of the main text.

Table S 9: Multiple comparisons of means, for the importance of predictors used in ODM

Linear hypothesis	Estimate	SE	z value	Pr(>|z|)
**mTCM - TSeasSD == 0**	**-0.035**	**0.011**	**-3.108**	**0.016**
MTWQ - TSeasSD == 0	-0.319	0.011	-28.343	<0.001
MTDQ - TSeasSD == 0	-0.469	0.011	-41.759	<0.001
RainWQ - TSeasSD == 0	-0.368	0.011	-32.770	<0.001
MTWQ - mTCM == 0	-0.284	0.011	-25.235	<0.001
MTDQ - mTCM == 0	-0.434	0.011	-38.651	<0.001
RainWQ - mTCM == 0	-0.333	0.011	-29.662	<0.001
MTDQ - MTWQ == 0	-0.151	0.011	-13.416	<0.001
RainWQ - MTWQ == 0	-0.050	0.011	-4.426	<0.001
RainWQ - MTDQ == 0	0.101	0.011	8.990	<0.001

Fig. S 5 shows the probability of orchards occurrence (*p*), for a Maxent model built exclusively with *Minimum Temperature of Coldest Month* (mTCM). Values of *mTCM* are taken from present conditions. Greatest *p* is predicted around 1.5 °C. The red line shows the mean from 10 Maxent models, the blue shade shows 1 standard deviation from the mean.

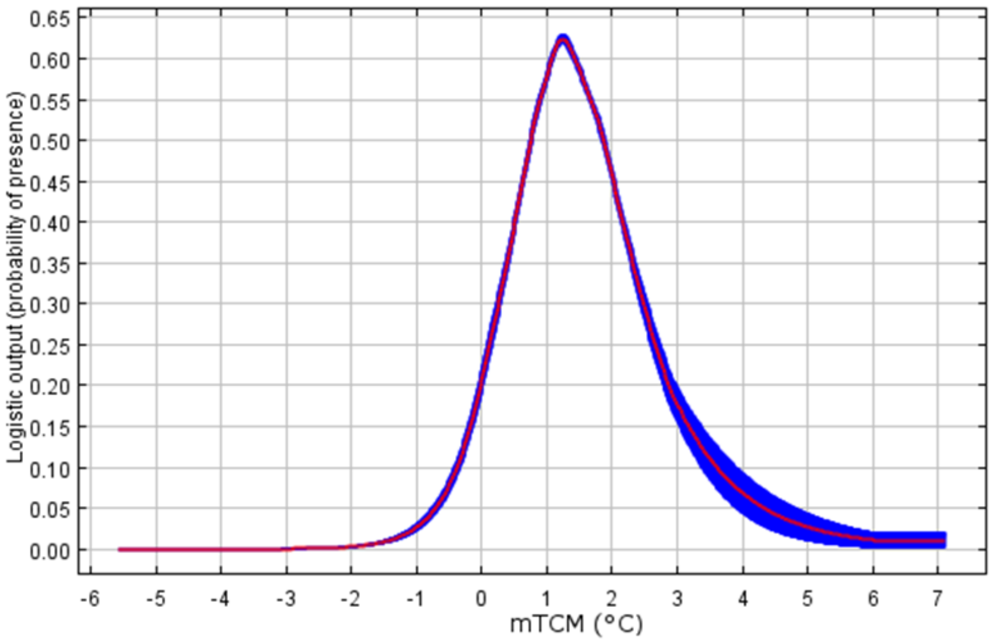

Figure S 5: Dependency of crop probability of occurrence on Minimum Temperature of Coldest Month

Future projections

The two histograms in Fig. S 6 show current and future distribution of Spearman's correlations between orchards extent and probability of occurrence (*p*), based on 9999 samples with replacement. The dashed lines indicate the observed correlation in each period. Probability of occurrence was estimated using climatic predictors, with Maxent. From the position of the dashed line we conclude that the correlation between crop extent and *p* is significantly different than what it could be expected by chance alone. In particular, there is a positive correlation between crop extent and *p* in the present ($\rho = 0.153$), and a negative correlation ($\rho = -0.233$) between the current crop extent and the future *p*.

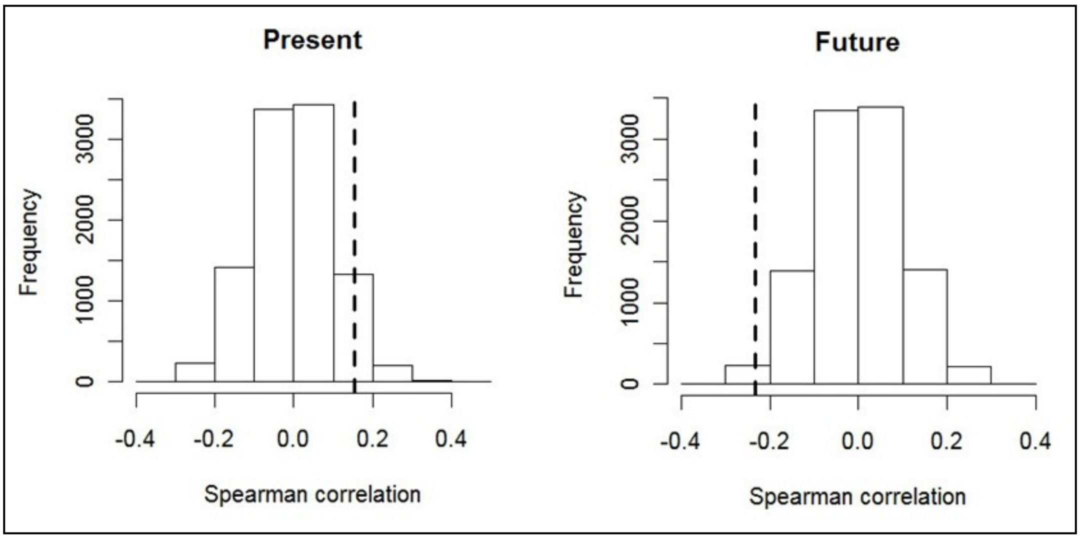

Figure S 6: Observed and simulated correlation between orchards extent and probability of occurrence for present and future climatic conditions

Fig. S 7 shows the predicted pollinator availability (PA) currently available to orchards. PA is used as a proxy for pollination service, and measured with a relative index from 0 to 1. PA is mapped from red to green using intervals. Red is used only to indicate areas where PA is predicted to be 0 (i.e. where pollinators are predicted to be absent). The chart shows the frequency of the predicted PA, following the same intervals used for the map. Frequency is measured as number of grid cells with a certain value of PA. The intervals are based on a common scale, used for Figs S 7 to S 9.

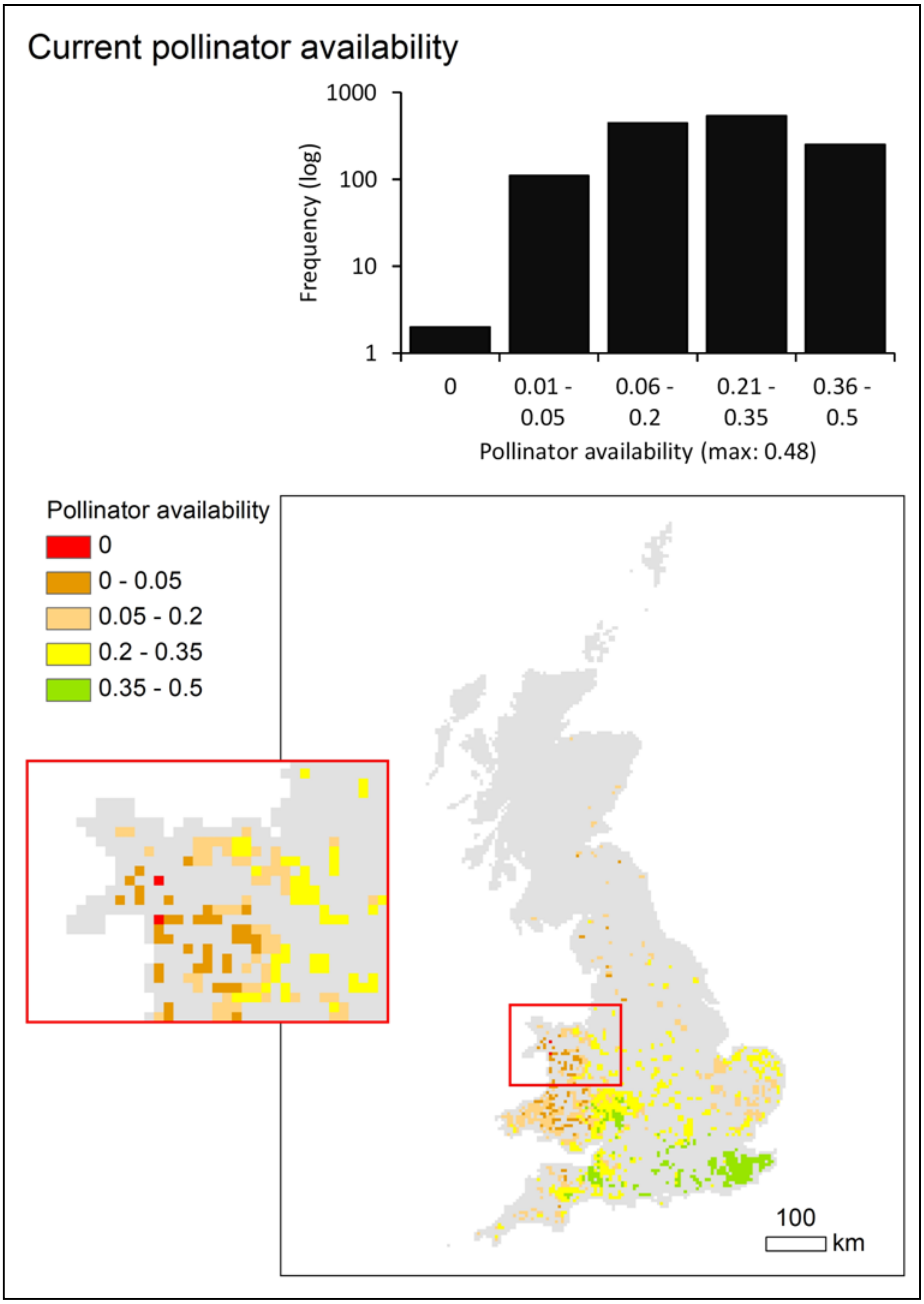

Figure S 7: Current pollinator availability to orchards

Fig. S 8 shows the predicted future PA available to orchards, in areas identified as the most suitable to crop growth, based on future climatic conditions (i.e. M2050 scenario). Red is used to map areas where PA is predicted to be 0, due to absence of wild pollinators. The chart shows the frequency of the predicted PA, following the same intervals used for the map. Frequency is measured as number of grid cells with a certain value of PP. The intervals are based on a common scale, used for Figs S 7 and S 9.

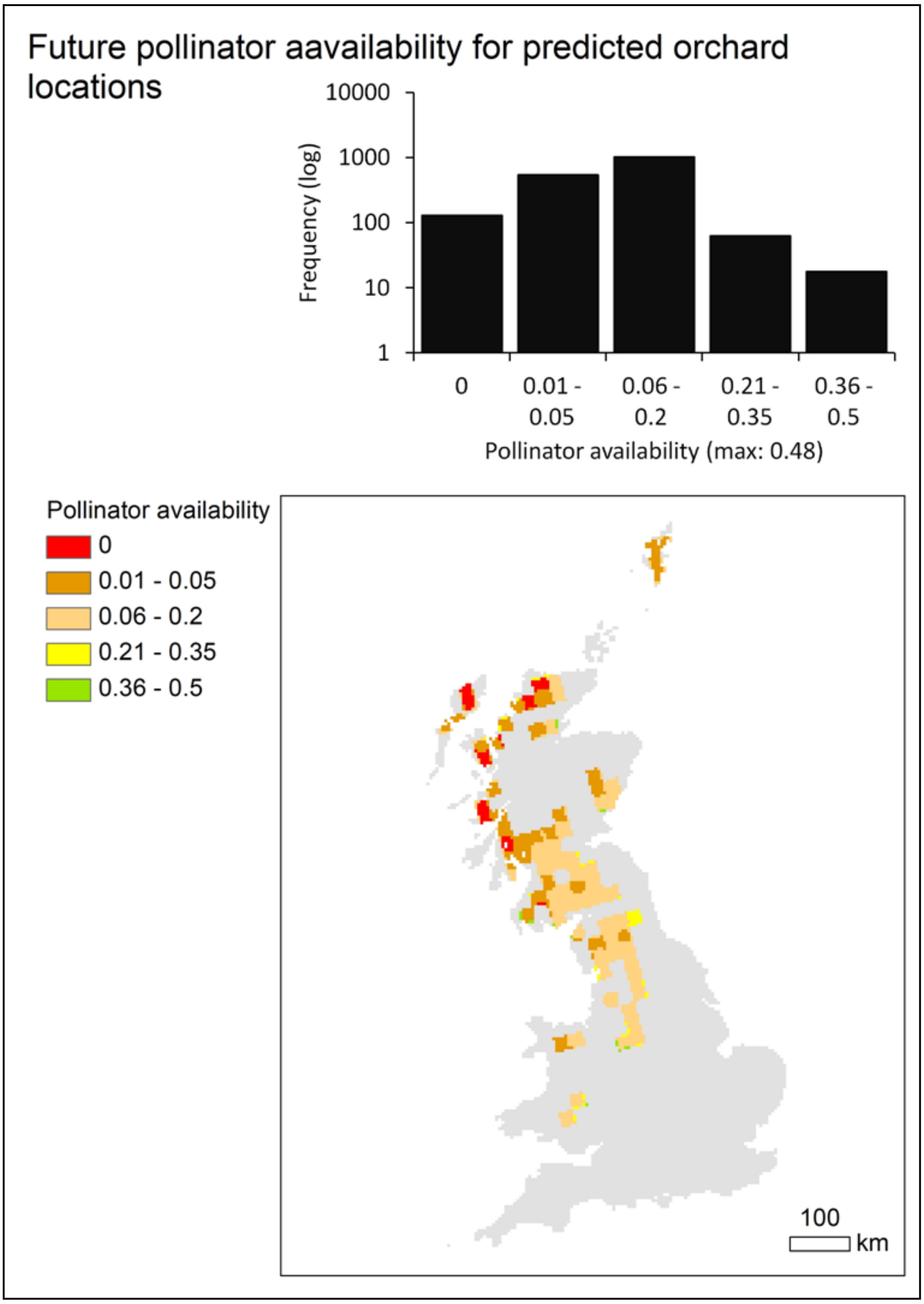

Figure S 8: Future pollinator availability in areas predicted to have the greatest suitability for orchards under future climatic conditions

Fig. S 9 shows the predicted PA where orchards are currently grown, based on future climatic conditions (i.e. M2050 scenario). PA is mapped using interval classes, following the same scale adopted for Figs S 7 and S 8. The map suggests that, if orchards persisted where they are currently planted, they would all receive some pollination service. Green shades are used to map areas with greater PA. The chart shows the frequency of the predicted PA, following the same intervals used for the map. Frequency is measured as number of grid cells with a certain value of PA. The intervals are based on a common scale, used also for Fig. S 7 and S 8.

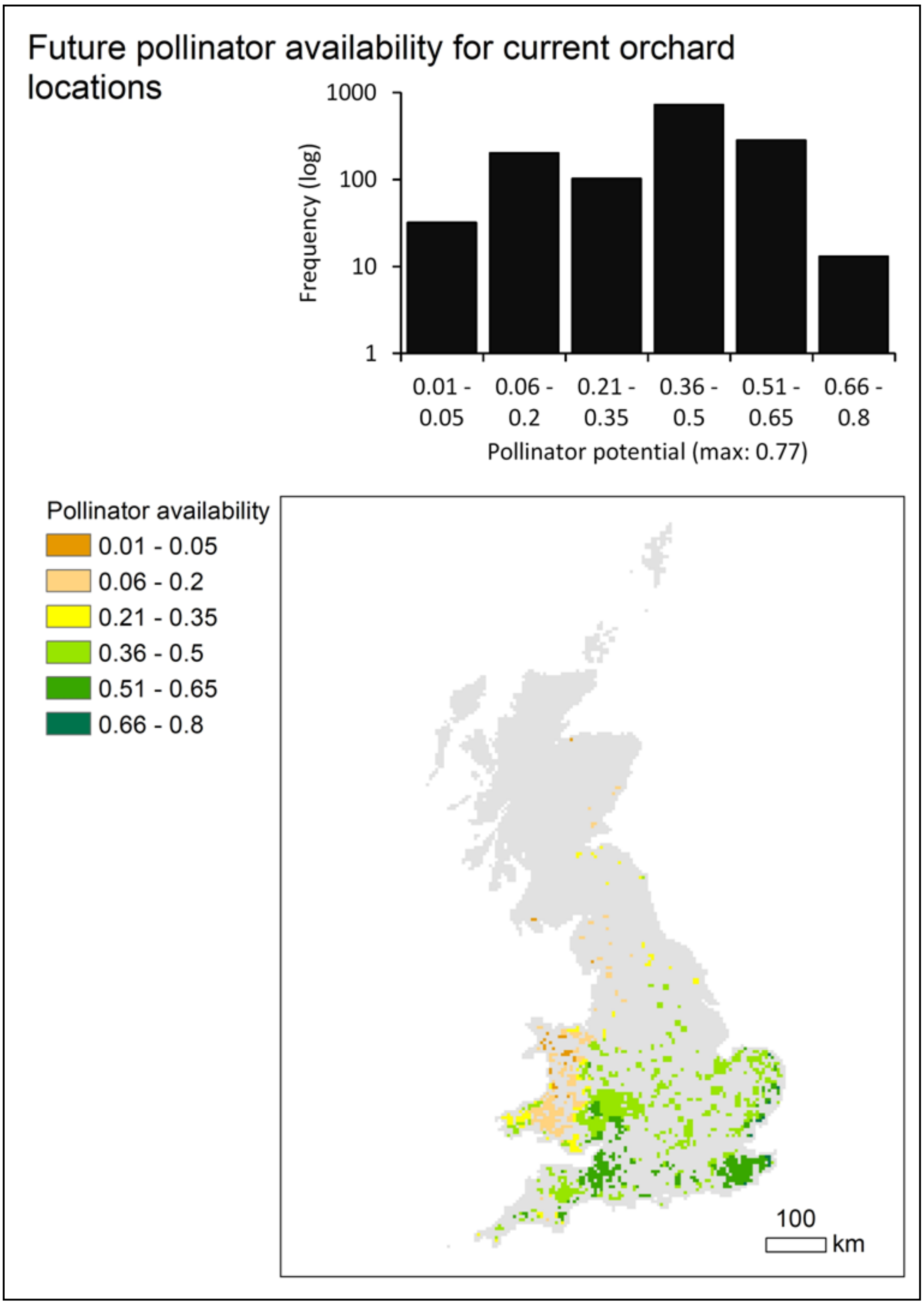

Figure S 9: Future pollinator potential predicted where orchards are currently planted

References

Franklin J, Davis FW, Ikegami M, Syphard AD, Flint LE, Flint AL, Hannah L (2013) Modeling plant species distributions under future climates: how fine scale do climate projections need to be? *Global Change Biology,* **19**, 473-483.

Hothorn T, Bretz F, Westfall P, Heiberger RM, Schuetzenmeister A (2013) multcomp: Simultaneous Inference in General Parametric Models.

Jolliffe IT (1972) Discarding Variables in a Principal Component Analysis. I: Artificial Data. *Applied Statistics,* **2**, 160-173.

Jolliffe IT (1973) Discarding Variables in a Principal Component Analysis, II: Real Data. *Applied Statistics,* **22**, 21-31.

Murphy JM, Sexton DMH, Jenkins GJ *et al.* (2010) UK Climate Projections Science Report: Climate change projections., Met Office Hadley Centre. Exeter, UK.

Nakićenović N, Alcamo J, Davis G *et al.* (2000) *IPCC Special Report on Emissions Scenarios (SRES)*, Cambridge University Press.

Phillips SJ, Dudík M, Elith J, Graham CH, Lehmann A, Leathwick J, Ferrier S (2009) Sample selection bias and presence-only distribution models: implications for background and pseudo-absence data. *Ecological Applications,* **19**, 181-197.

Polce C, Termansen M, Aguirre-Gutiérrez J *et al.* (2013) Species Distribution Models for Crop Pollination: A Modelling Framework Applied to Great Britain. *PLoS ONE,* **8**, e76308.

Sork VL, Davis FW, Westfall R, Flint A, Ikegami M, Wang H, Grivet D (2010) Gene movement and genetic association with regional climate gradients in California valley oak (Quercus lobata Nee) in the face of climate change. *Molecular Ecology,* **19**, 3806-3823.

Termansen M, Mcclean CJ, Preston CD (2006) The use of genetic algorithms and Bayesian classification to model species distributions. *Ecological Modelling,* **192**, 410-424.

Thuiller W (2004) Patterns and uncertainties of species' range shifts under climate change. *Global Change Biology,* **10**, 2020-2027.

Warren R, Vanderwal J, Price J *et al.* (2013) Quantifying the benefit of early climate change mitigation in avoiding biodiversity loss. *Nature Climate Change,* **3**, 678-682.
